# Large mid-upper arm circumference is associated with metabolic syndrome in middle-aged and elderly individuals: a community-based study

**DOI:** 10.1186/s12902-020-00559-8

**Published:** 2020-06-03

**Authors:** Jie Shi, Zhen Yang, Yixin Niu, Weiwei Zhang, Xiaoyong Li, Hongmei Zhang, Ning Lin, Hongxia Gu, Jie Wen, Guang Ning, Li Qin, Qing Su

**Affiliations:** 1 Department of Endocrinology, Xinhua Hospital Chongming Branch, Shanghai Jiaotong University School of Medicine, 25 Nanmen Road, Shanghai, 202150 China; 2 Department of Endocrinology, Xinhua Hospital, Shanghai Jiaotong University School of Medicine, Shanghai, 200092 China; 3 Institute of Endocrinology and Diabetes, Department of Endocrinology and Metabolism, Huashan Hospital, Fudan University, Shanghai, 200040 China; 4 Shanghai Institute of Endocrinology and Metabolism, Department of Endocrine and Metabolic Diseases, Shanghai Clinical Center for Endocrine and Metabolic Diseases, Ruijin Hospital, Shanghai Jiaotong University School of Medicine, Shanghai, 200025 China

**Keywords:** Metabolic syndrome, Mid-upper arm circumference, Central obesity, Subcutaneous fat

## Abstract

**Background:**

The mid-upper arm circumference (MUAC) is a proxy for subcutaneous fat in the upper body and is a reliable screening measure for identifying individuals with abnormal regional fat distribution. The purpose of this study was to evaluate the association between MUAC and metabolic syndrome (MetS) in middle-aged and elderly individuals.

**Methods:**

We measured the MUAC in a cross-sectional sample with a total of 9787 subjects aged 40 years and older. The measurement of MUAC is performed on the right arm using a non-elastic tape held midway between the acromion and the olecranon processes in duplicate, with the arm hanging loosely at the side of the body. The MetS was defined according to the Joint Statement of the International Diabetes Federation Task Force on Epidemiology and Prevention.

**Results:**

MUAC was positively correlated with waist circumference (r = 0.437, *P* < 0.001), BMI (r = 0.334, P < 0.001), fasting insulin (r = 0.348, *P* < 0.001), HOMA-IR (r = 0.134, *P* < 0.001), triglycerides (r = 0.138, P < 0.001), SBP (r = 0.124, P < 0.001), and DBP (r = 0.123, P < 0.001), and inversely correlated with adiponectin (r = − 0.147, P < 0.001) and HDL-cholesterol (r = − 0.176, P < 0.001) after adjusting for age and gender. Compared with the lowest quartile group, the odds ratios were substantially higher for MetS (OR 1.77; 95% CI 1.51–2.09, P for trend< 0.001) in the highest MUAC quartile group after adjustment for potential cofounder.

**Conclusion:**

Large mid-upper arm circumference is significantly associated with metabolic syndrome in middle-aged and elderly individuals.

## Background

Metabolic syndrome (MetS), which contains a cluster of metabolic abnormalities including central obesity, hypertension, dysglycemia, and dyslipidemia that together culminate in the increased risk of cardiovascular disease (CVD) and diabetes, is a major global public health problem [1]. Of note, the prevalence of MetS is up to 30% in middle-aged and elderly people in China [2, 3]. Given the continuous increase of the aging population in China, the high prevalence of MetS is a concerning observation that should be put on the agenda.

It is well established that different body fat distribution exist significant differences not only in free fatty acid (FFA) storage but also in FFA release [4]. Moreover, compared with the lower-body subcutaneous fat, the upper-body subcutaneous adipose tissue exhibited more active lipolysis [5, 6]. Numerous convincing evidence demonstrated that upper-body subcutaneous fat is significantly associated with increased visceral fat and involved in metabolic disorders independent of body mass indexes [7, 8]. Previous studies have revealed that systemic FFA is primarily derived from upper-body subcutaneous fat [5, 9], indicating that excessive upper arm fat depot may be a contributor to metabolic abnormality. On the one hand, the transfer of excess FFA from systemic adipose tissue lipolysis to liver tissue will result in excessive fat accumulation in the liver. On the other hand, elevated FFA levels also provoke VLDL-triglyceride production, insulin resistance, and inflammation, further increases the risk of metabolic disorders [10]. Furthermore, it has been demonstrated that dysregulation of fatty acid disposition, with ectopic lipid accumulation in non-adipose cells, is a major factor contributing to the development of MetS [11].

More recently, several studies have reported that mid-upper arm circumference (MUAC), a feasible and valid screening tool for identifying subjects with abnormal distribution of upper-body subcutaneous fat, was positively associated with higher risks of cardiometabolic disorders and subclinical atherosclerosis independent of waist circumference and BMI [12]. To date, however, much less is known about the relationship between MUAC and MetS among Chinese individuals. Thus, the purpose of this study was to evaluate the relationship between MUAC and MetS among Chinese middle-aged and elderly individuals.

## Methods

### Study population

The study is a part of the Risk Evaluation of cAncers in Chinese diabeTic Individuals: A lONgitudinal (REACTION) study, a community-based study conducted among 259,657 Chinese individuals aged 40 years and older [13]. REACTION study performed in 25 communities across mainland China, from 2011 to 2012. The study design and methods have been described previously in detail [13–15]. The data presented in this article are based on the baseline survey of subsamples from the Chongming District, Shanghai, China. A total of 9930 eligible subjects participated in the study. After excluding individuals with missing data about MUAC (*n* = 143), 9787 subjects (3156 men and 6631 women) were eventually included in the present analysis. The study protocol was approved by the Ethics Committee of Xinhua Hospital Affiliated to Shanghai Jiaotong University School of Medicine. Written informed consent was obtained from all participants.

### Data collection

A standardized questionnaire was applied by trained physicians to collect essential information, including sex, age, lifestyle factors, educational attainment, physical activity, and previous medical history. Anthropometric measurements were collected by certified physicians using standard protocols in duplicate base on the National Health and Nutrition Examination Survey (NHANES) Anthropometry Procedures Manual [16]. The measurement of MUAC is performed on the right arm using a non-elastic tape held midway between the acromion and the olecranon processes, with the arm hanging loosely at the side of the body. Waist circumference was measured with a non-elastic tape held midway between the lower rib margin and the iliac crest at the end of a gentle expiration. Blood pressure (BP) was measured with an automated electronic device (OMRON Model1 Plus; Omron Company, Kyoto, Japan). Overweight was defined as body mass index (BMI) ≥ 24 kg/m^2^, central obesity was defined as waist circumference ≥ 85 cm for men and ≥ 80 cm for women.

### Biochemical measurements

Peripheral venous blood samples were collected after an overnight fast for at least 10 h. The plasma glucose level was measured by the glucose oxidase method (ADVIA-1650 Chemistry System, Bayer, Leverkusen, Germany). We used an automatic analyzer (Hitachi 7080; Tokyo, Japan) to measured low-density lipoprotein (LDL) cholesterol, high-density lipoprotein (HDL) cholesterol, total cholesterol, and triglycerides. Hemoglobin A1c was determined by the HPLC method (BIO-RAD, D10, CA). Fasting insulin was measured by RIA (Linco Research, St. Charles, MO) and insulin resistance was evaluated using the homeostasis model of assessment for insulin resistance (HOMA-IR).

### Definition of MetS

The MetS and metabolic risk factors were defined based on the Joint Statement of the World Heart Federation; National Heart, Lung, and Blood Institute; American Heart Association; International Diabetes Federation Task Force on Epidemiology and Prevention; International Atherosclerosis Society; and International Association for the Study of Obesity [1]. MetS was defined as having ≥3 of the following metabolic risk factors: (1) central obesity (waist circumference ≥ 85 cm in men or ≥ 80 cm in women), (2) triglycerides ≥1.7 mmol/L, (3) HDL cholesterol < 1.0 mmol/L in men or < 1.3 mmol/L in women, (4) systolic blood pressure (SBP) ≥ 130 and/or diastolic blood pressure (DBP) ≥ 85 mmHg or current use of antihypertensive agents, (5) fasting plasma glucose ≥5.6 mmol/L, previous diagnosis of type 2 diabetes or use of antidiabetic agents.

### Statistical analysis

Continuous variables with normal distribution were expressed as means ± SD, variables with skewed distribution were shown as median (interquartile range) and log-transformed to approximate normality before analysis. Categorical variables were expressed by frequency and percentage. The subjects were divided into four groups according to the MUAC quartiles. For comparisons between groups, we performed one-way ANOVA for continuous variables with normal distributions and nonparametric tests for continuous variables with highly skewed distributions. The Chi-squared test was applied to compare categorical variables. Correlation coefficients between MUAC and metabolic features were calculated by partial correlation analysis after adjusted age and gender. Multivariate logistic regression analyses were used to test odds ratios (ORs) and 95% confidence intervals (CIs) of Mets for each MUAC quartile compared with the lowest quartile (Q1) group. Model 1 was adjusted for age and gender; model 2 was adjusted for the variables in model 1 plus smoking, alcohol drinking, educational attainment, physical activity, and self-reported CVD; model 3 was adjusted for the variables in model 2 plus CRP, adiponectin, and HOMA-IR; model 4 was adjusted for the variables in model 3 plus BMI. Data management and statistical analysis were performed with SPSS (version 23.0). *P* < 0.05 was considered statistically significant.

## Results

### Characteristics of participants according to MUAC quartiles

The mean of MUAC was 29.24 cm for males and 28.41 cm for females (*P* < 0.001), respectively. The individuals were divided into four groups based on the quartiles of MUAC. When analyzed by quartiles of MUAC levels, as summarized in Table 1, the subjects with larger MUAC were more likely to be smokers, alcohol drinkers, and comorbidities including hypertension, hyperlipidemia, diabetes (all *P* < 0.05). With regard to metabolic parameters, the individuals in the higher MUAC quartiles showed higher levels of SBP, DBP, BMI, waist circumference or waist-to-hip ratio, fasting glucose, insulin, HOMA-IR, CRP, triglycerides (all *P* < 0.001), and LDL cholesterol (*P* = 0.003). Conversely, the subjects with larger MUAC displayed lower adiponectin and HDL cholesterol levels (both P < 0.001). However, elevated MUAC exhibited no association with the levels of total cholesterol in this study.

**Table 1:** Characteristics of study participants according to MUAC quartiles

Characteristics	Q1 (*n* = 2524)	Q2 (*n* = 2409)	Q3 (*n* = 2427)	Q4 (n = 2427)	*P* value for trend
	≤26.70	26.71–28.60	28.61–30.50	≥30.51	
MUAC (cm)	24.93 ± 1.53	27.78 ± 0.56	29.61 ± 0.53	32.53 ± 1.72	< 0.001
Age (years) ^b^	55.59 ± 8.21	56.06 ± 7.81	56.53 ± 7.62	56.47 ± 7.59	< 0.001
Male (%) ^b^	24.0	29.1	37.1	39.1	< 0.001
Smoking (yes, %)	14.3	17.2	18.1	19.9	< 0.001
Alcohol (yes, %)	18.1	24.5	26.0	28.1	< 0.001
Educational attainment (%)					
0–6 years	20.7	21.7	21.8	25.4	0.003
7–9 years	50.0	49.1	48.6	48.2	
≥ 10 years	29.3	29.2	29.6	26.4	
Physical activity (%)					
Low	70.8	72.5	71.6	72.2	0.281
Moderate	21.4	19.6	21.2	19.7	
High	7.8	7.9	7.2	8.1	
SBP (mm Hg)	126.96 ± 19.00	129.72 ± 18.42	132.23 ± 17.76	134.55 ± 18.94	< 0.001
DBP (mm Hg)	79.02 ± 9.51	80.29 ± 9.71	81.13 ± 9.50	82.50 ± 9.40	< 0.001
BMI (kg/m^2^)	22.79 ± 2.90	24.47 ± 2.48	25.46 ± 2.61	26.94 ± 3.08	< 0.001
Waist circumference (cm)	78.38 ± 11.41	82.94 ± 8.49	86.54 ± 8.75	91.11 ± 8.77	< 0.001
Waist to hip ratio	0.86 ± 0.08	0.87 ± 0.09	0.89 ± 0.17	0.90 ± 0.07	< 0.001
Fasting glucose (mmol/L)	6.20 ± 1.82	6.23 ± 1.73	6.33 ± 1.65	6.38 ± 1.52	< 0.001
2-h glucose (mmol/L)	8.30 ± 4.01	8.61 ± 3.94	8.91 ± 3.79	9.18 ± 3.73	< 0.001
Insulin (mU/L) ^c^	6.25 (3.90–7.20)	7.18 (4.50–8.50)	7.75 (5.20–9.20)	9.40 (6.10–11.50)	< 0.001
HOMA-IR ^c^	1.70 (0.98–1.99)	2.00 (1.22–2.45)	2.22 (1.38–2.68)	2.70 (1.65–3.38)	< 0.001
CRP (ug/ml)	4.79 ± 5.74	4.94 ± 5.85	5.13 ± 6.12	5.24 ± 6.27	< 0.001
Adiponectin (ug/ml)	12.34 (7.92–15.67)	10.93 (6.72–13.15)	8.17 (5.24–11.33)	6.29 (4.78–9.75)	< 0.001
Triglycerides (mmol/L) ^c^	1.44 (0.83–1.66)	1.67 (0.94–1.94)	1.83 (1.03–2.13)	1.96 (1.12–2.34)	< 0.001
Total cholesterol (mmol/L)	4.64 ± 1.05	4.62 ± 1.03	4.65 ± 1.04	4.67 ± 1.03	0.291
LDL cholesterol (mmol/L)	2.58 ± 0.78	2.59 ± 0.76	2.63 ± 0.77	2.65 ± 0.76	0.003
HDL cholesterol (mmol/L)	1.32 ± 0.34	1.23 ± 0.32	1.19 ± 0.30	1.16 ± 0.28	< 0.001
Comorbidities (%)					
Hypertension	20.8	25.0	31.4	36.9	< 0.001
Hyperlipidemia	2.8	3.4	5.8	6.2	< 0.001
Diabetes	8.8	9.3	10.8	10.8	0.036
CVD ^d^	2.6	3.9	3.2	4.1	0.014

^a^ Data are means ± SD, median (interquartile range), or percentage; P value was calculated after adjustment for age and gender. ^b^ Not adjusted for itself. ^c^ These variables were log transformed before analysis. ^d^ Self-reported CVD including stroke and coronary heart disease

### Association between MUAC and MetS

Partial correlation analysis showed that MUAC has the strongest correlation with waist circumference after adjusting for age and gender (Table 2). Additionally, MUAC was also strongly correlated with BMI and insulin.

**Table 2:** Partial correlation coefficients among MUAC and other clinical parameters

Variable	MUAC	
	r	P
BMI	0.334	< 0.001
Waist circumference	0.437	< 0.001
SBP	0.124	< 0.001
DBP	0.123	< 0.001
Fasting plasma glucose	0.045	0.043
2-h post-loading glucose	0.074	< 0.001
Insulin	0.348	< 0.001
HOMA-IR	0.134	< 0.001
CRP	0.110	0.008
Adiponectin	−0.147	< 0.001
HDL cholesterol	−0.176	< 0.001
LDL cholesterol	0.024	0.016
Triglycerides	0.138	< 0.001

All correlation coefficients were calculated after adjustment for age and gender

**Fig. 1:**
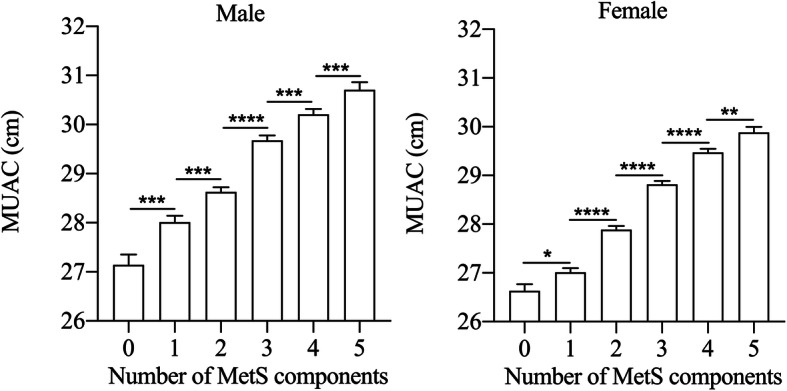
MUAC according to the number of MetS components. Data are shown as the means ± SEM. The mean values of MUAC for those with none to five components were 27.14, 28.01, 28.63, 29.70, 30.21, 30.71 cm for males, and 26.62, 27.00, 27.83, 28.79, 29.43, 29.8 cm for females, respectively. *: *P* < 0.05; **: *P* < 0.01; ***: *P* < 0.001; ****: *P* < 0.0001. MUAC, mid-upper arm circumference; MetS, metabolic syndrome

The prevalence of MetS, overweight, and central obesity raised sharply from the small MUAC group to the large MUAC group both in men and women (All P for trend < 0.001). In addition, women seem to have a higher prevalence of MetS and central obesity compared with men. Furthermore, with the accumulation of MetS components, MUAC was gradually increased both in males and females (Fig. 1). It is worth noting that as the components of MetS increased from none to five, the relationship between MUAC and MetS was statistically significant, both in subjects with Mets (number of MetS components ≥3) and the subjects without MetS (number of MetS components < 3). On this ground, MUAC may be directly or indirectly related to each MetS component.

As is shown in Table 3, the ORs for MetS and its components were significantly higher with increasing MUAC quartiles. In the largest MUAC quartile, the ORs (95% CIs) were 5.71 (4.97–6.56) for MetS, 14.70 (12.49–17.31) for central obesity, 2.23 (1.90–2.62) for elevated triglycerides, 2.11 (1.86–2.40) for elevated blood pressure, 2.06 (1.79–2.37) for reduced HDL cholesterol, 1.54 (1.35–1.76) for elevated fasting glucose after adjusting for age, gender, smoking, alcohol consumption, educational attainment, physical activity, CRP, adiponectin, and self-reported CVD (referencing to 1.00) (Model 2, all P for trend < 0.001). Further adjustment for HOMA-IR, CRP, and adiponectin, the OR was 3.24 (Model 3, 95% CI 2.79–3.76, P for trend < 0.001) for MetS in the highest MUAC quartile. Moreover, the correlation between MUAC and MetS was still statistically significant by additional adjustment for BMI (Model 4, OR 1.77; 95% CI 1.51–2.09; P for trend < 0.001).

**Table 3:** Adjusted ORs (95% CI) of MetS and its components according to the quartiles of MUAC

	ORs (95% CI)				P value for trend
	Q1	Q2	Q3	Q4	
MetS					
Model 1^a^	1.00	1.79 (1.60–2.02)	3.28 (2.91–3.70)	5.77 (5.08–6.54)	< 0.001
Model 2^b^	1.00	1.77 (1.56–2.01)	3.25 (2.85–3.70)	5.71 (4.97–6.56)	< 0.001
Model 3^c^	1.00	1.55 (1.35–1.78)	2.49 (2.17–2.86)	3.24 (2.79–3.76)	< 0.001
Model 4^d^	1.00	1.23 (1.07–1.42)	1.68 (1.45–1.95)	1.77 (1.51–2.09)	< 0.001
Central obesity					
Model 1^a^	1.00	2.50 (2.22–2.81)	5.67 (5.00–6.43)	15.31 (13.17–17.81)	< 0.001
Model 2^b^	1.00	2.47 (2.18–2.81)	5.52 (4.82–6.31)	14.70 (12.49–17.31)	< 0.001
Model 3^c^	1.00	2.29 (2.00–2.61)	4.69 (4.08–5.40)	10.57 (8.93–12.52)	< 0.001
Model 4^d^	1.00	1.41 (1.20–1.65)	2.03 (1.71–2.40)	3.46 (2.81–4.25)	< 0.001
Elevated blood pressure					
Model 1^a^	1.00	1.27 (1.13–1.43)	1.66 (1.48–1.87)	2.15 (1.91–2.42)	< 0.001
Model 2^b^	1.00	1.26 (1.11–1.43)	1.64 (1.45–1.86)	2.11 (1.86–2.40)	< 0.001
Model 3^c^	1.00	1.18 (1.03–1.34)	1.45 (1.27–1.65)	1.65 (1.44–1.88)	< 0.001
Model 4^d^	1.00	1.04 (0.91–1.18)	1.16 (1.02–1.33)	1.17 (1.01–1.35)	< 0.001
Elevated triglycerides					
Model 1^a^	1.00	1.44 (1.23–1.69)	1.81 (1.55–2.11)	2.34 (2.02–2.72)	< 0.001
Model 2^b^	1.00	1.39 (1.17–1.65)	1.81 (1.54–2.14)	2.23 (1.90–2.62)	< 0.001
Model 3^c^	1.00	1.26 (1.06–1.50)	1.49 (1.26–1.77)	1.52 (1.28–1.80)	< 0.001
Model 4^d^	1.00	1.21 (1.02–1.45)	1.36 (1.14–1.64)	1.40 (1.17–1.67)	< 0.001
Reduced HDL cholesterol					
Model 1^a^	1.00	1.45 (1.27–1.66)	1.70 (1.49–1.93)	2.18 (1.91–2.48)	< 0.001
Model 2^b^	1.00	1.36 (1.18–1.58)	1.68 (1.45–1.94)	2.06 (1.79–2.37)	< 0.001
Model 3^c^	1.00	1.29 (1.11–1.50)	1.50 (1.30–1.74)	1.68 (1.45–1.95)	< 0.001
Model 4^d^	1.00	1.23 (1.06–1.43)	1.38 (1.19–1.61)	1.46 (1.24–1.71)	< 0.001
Elevated fasting glucose					
Model 1^a^	1.00	1.28 (1.13–1.45)	1.48 (1.32–1.72)	1.51 (1.33–1.73)	< 0.001
Model 2^b^	1.00	1.28 (1.12–1.47)	1.51 (1.04–1.34)	1.54 (1.35–1.76)	< 0.001
Model 3^c^	1.00	1.23 (1.08–1.41)	1.41 (1.23–1.61)	1.38 (1.20–1.57)	< 0.001
Model 4^d^	1.00	1.14 (0.99–1.32)	1.15 (1.00–1.32)	1.25 (1.09–1.44)	< 0.001

^a^ Model 1 was adjusted for age and gender. ^b^ Model 2 was further adjusted for smoking, alcohol drinking, educational attainment, physical activity, and self-reported CVD. ^c^ Model 3 was further adjusted for CRP, adiponectin, and HOMA-IR. ^d^ Model 4 was further adjusted for BMI. Q, quartile. Q1 is the reference group. Multivariate logistic regression models were used to estimate the odds ratios (ORs) with corresponding 95% confidence intervals (CIs) for MetS and its components. The final model is statistically significant (χ^2^ = 34.927, P < 0.001)

## Discussion

We observed a significant positive association between MUAC and MetS in a large-scale population study. Furthermore, we found that MUAC is strongly correlated with waist circumference, lipid parameters, blood pressure, and insulin, which indicated that abnormal local fat depot is a potential screening index for identifying metabolic disorders.

BMI and waist circumference are common screening measures for identifying individuals with abnormal distribution of body fat. Nevertheless, BMI cannot provide accurate information about the local distribution of body fat and it is difficult to obtain height and weight for patients who cannot stand. As for waist circumference, the deficiency of daily application lies in the big difference between preprandial and postprandial measurements. In view of the above reasons, MUAC began to show diagnostic value for assessing nutritional status. Compared to other anthropometric measurements, MUAC is not only easier to obtain, but also has other advantages such as being more accurate, convenient, and low-cost. Small MUAC has shown excellent performance in assessing malnutrition and predicting mortality both in children [17] and older individuals [18, 19]. More recently, large MUAC has been recognized as a valid tool for detecting overweight and obesity in children and adolescents [20, 21]. However, the study about whether MUAC is associated with obesity-related metabolic abnormality, such as MetS, is scarce. Currently, we found that MUAC, as a proxy of upper-body subcutaneous adipose, was positively associated with MetS. Moreover, consistent with previous studies [12], we found large MUAC also tightly correlated with central obesity, elevated blood pressure, and low HDL cholesterol.

The abnormal accumulation of fat affects adipose tissue metabolic capacities, endocrine, and immune function and leads to altered production of lipid mediators, adipokines, pro- or anti-inflammatory cytokines, and impaired signaling pathways that contribute to obesity-related metabolic abnormality [22]. Obesity increases the flux of fatty acids from adipose tissue to peripheral tissues [23]. The increase in FFAs derived from adipose tissue is mainly mediated by the resistance of adipose tissue to the anti-lipolytic action of insulin [24]. There is compelling evidence that abnormally increased visceral fat is a maker of excessive systemic FFAs release [25], but it is worth noting that it is upper-body subcutaneous fat released the majority of FFAs [26]. Large MUAC means excessive subcutaneous fat accumulation, which contributes a greater portion of the fatty acids released into the circulation. Circulating FFAs is a crucial mediator in the development of metabolic disorders [27]. Elevated plasma FFAs induce insulin resistance, inflammation, and increase the synthesis and ectopic deposition of triglycerides [28–31]. Concomitantly, excessive FFAs also affect glucose metabolism by inhibiting glucose uptake, oxidation, glycogen synthesis, and increasing output hepatic glucose [32]. Additionally, increased FFAs can trigger oxidative stress which is an early instigator of MetS [33], and endoplasmic reticulum stress which intersects with many different inflammatory and stress signaling pathways by unfolded protein response [34–36]. The excess FFAs release derived from excess accumulation of arm subcutaneous adipose might be a potential mechanism to partly explain the correlation between MUAC and MetS.

Moreover, the adipose tissue is not only a depot of excess energy but also a highly active metabolic endocrine organ that secretes numerous biologically active molecules, which are collectively termed adipokines [37]. When adipose tissue expands, the capacity of adipocytes to function as endocrine cells and secrete various adipokines is altered in individuals with obesity and MetS [33, 38]. These abnormal levels of adipokine are linked to insulin resistance, impaired triglyceride storage and increased fatty acids in circulation [39]. Furthermore, as fat accumulation, substantial infiltration of immune cells occurs, and there is a specific crown-like disposition of macrophages around single necrotic adipocytes in obese people [40] and subjects with MetS [38]. Subsequently, proinflammatory pathways were activated, and certain proinflammatory cytokines and chemokines were overflowed that result in low-grade inflammation and insulin resistance [22, 38]. In line with the previous study [12], our findings also observed that MUAC is positively correlated with CRP. Overall, adipose dysfunctions, inflammation, and stress linking mid-upper arm obesity to insulin resistance and MetS.

To our knowledge, this is the first study to evaluate the association between MUAC and Mets among large-scale middle-aged and older people. The major strength of this study is the analysis based on a large sample. Potential covariates were strictly controlled in the analysis, so as to eliminated the possibility of residual confounding effects.

This study has several limitations. For one thing, we did not measure the tissue composition of the mid-upper arm. The use of MUAC to evaluate the mid-upper arm subcutaneous fat was a convenient and practical way but was unable to quantify the fat accumulation. Therefore, the amount and size of subcutaneous adipocyte and muscle fat are not clear. For another, due to the present study is a cross-sectional analysis, we cannot draw the causality from our findings. Additionally, it is still unclear whether our findings in middle-aged/older Chinese subjects can be generalized to younger populations or individuals of other ethnicities.

In brief, our study observed that MUAC is positively associated with MetS even after adjustment for potential covariates. These findings provide a novel insight into the association between upper-body obesity and MetS, and a potential screening tool for identifying individuals with MetS. Further researches are necessary to explore the underlying pathophysiological mechanism of the relationship between mid-upper arm subcutaneous fat and MetS.

## Conclusion

In summary, in this population-based study, there is a significantly positive association between MUAC and MetS in Chinese middle-aged and elderly individuals. Our results indicated that MUAC in the routine clinical measurement is necessary to screen for metabolic disorders, including dyslipidemia, dysglycemia, and elevated blood pressure.

## Data Availability

All data generated and analyzed during the current study are included in the REACTION study. The data that support the findings of this study are available from the corresponding author on reasonable request. Inquiries for data access may be sent to the following e-mail address: yangzhen@xinhuamed.com.cn.

## Abbreviations

MUAC: Mid-upper arm circumference
MetS: Metabolic syndrome
FFAs: Free fatty acids
BMI: Body mass index
HDL: High-density lipoprotein
LDL: Low-density lipoprotein
CVD: Cardiovascular disease
T2D: Type 2 diabetes
HOMA-IR: Homeostasis model of assessment for insulin resistance
SBP: Systolic blood pressure
DBP: Diastolic blood pressure
OR: Odds ratios
CI: Confidence interval
CRP: C-reactive protein
SD: Standard deviation
